# Bone and tendon adaptations to 18-weeks rehabilitation and endurance and resistance training in postpartum British Servicewomen: a non-randomised controlled trial

**DOI:** 10.1038/s41598-026-51411-3

**Published:** 2026-05-02

**Authors:** Thomas J. O’Leary, Emma L. Bostock, Thea Jackson, Sophie L. Wardle, Kirsty J. Elliott-Sale, Craig Sale, Julie P. Greeves

**Affiliations:** 1 Army Health and Performance Research, Army Headquarters, Andover, UK; 2 Division of Surgery and Interventional Science, UCL, London, UK; 3 School of Science and Technology, Nottingham Trent University, Nottingham, UK; 4 Institute of Sport, Manchester Metropolitan University, Manchester, UK; 5 Norwich Medical School, University of East Anglia, Norwich, UK

**Keywords:** Bone modeling, Bone remodeling, DXA, Exercise, HRpQCT, Pregnancy, Anatomy, Diseases, Health care, Medical research

## Abstract

Women experience musculoskeletal changes following pregnancy that may increase musculoskeletal injury risk. This trial explored 18-weeks combined rehabilitation and endurance and resistance training (Intervention, *n* = 17) compared with standard-of-care (Control, *n* = 14) on areal bone mineral density (aBMD), tibial bone, and patella tendon properties in postpartum UK Servicewomen. Intervention received standard care plus pelvic floor and core strength exercises from week 1 to 18 (week 6 to 24 postpartum), and combined high-intensity interval and resistance training from week 6 to 18 (week 12 to 24 postpartum). Control received standard care. Whole-body DXA and tibial HRpQCT scans (4% and 30% sites) were taken at week 1 and 18. Patella tendon properties at rest and during isometric contractions were measured by ultrasound at weeks 1, 6, and 18. There was no effect of training on aBMD (*p* ≥ 0.100) but trunk (–1.6%) and spine (–2.3%) aBMD decreased from week 1 to 18, irrespective of group (*p* ≤ 0.021). There was no effect of training on tibial bone (*p* ≥ 0.151) or patella tendon properties (*p* ≥ 0.185). An 18-week rehabilitation and endurance and resistance training programme had no effect on bone or tendon properties in postpartum Servicewomen.

**Supplementary Information:**

The online version contains supplementary material available at 10.1038/s41598-026-51411-3.

## Introduction

Women experience musculoskeletal changes during pregnancy and following childbirth, including decreased areal bone mineral density (aBMD)^1^, altered bone microstructure in the peripheral skeleton^1^, and increased tendon elasticity^2^. These musculoskeletal changes can pose challenges to health and performance for those returning to high levels of physical activity after childbirth (e.g., athletes or Servicewomen)^3^. Military occupations are physically and psychologically demanding^4–9^ and musculoskeletal changes during and following pregnancy may increase the risk of musculoskeletal injuries and other musculoskeletal disorders in Servicewomen returning to work^10^. Data from the British Army show women are at increased risk of illness and musculoskeletal injury within the first year following childbirth compared with the year before childbirth^10^.

Servicewomen in the UK Armed Forces are entitled to maternity leave that includes 26 weeks full pay, followed by 13 weeks statutory pay, and then 13 weeks of unpaid leave; however, they can return after 2 weeks of compulsory maternity leave. Upon return to work, postpartum Servicewomen in the British Army are restricted from completing physically arduous activities (e.g., fitness tests, deployments, training) for at least 6 months. A total of 4.4% of all Servicewomen in the UK Armed Forces took maternity leave in 2023 with 24.5% returning to work by 27 weeks and 82.0% returning to work by 40 weeks^11^. Strategies that promote musculoskeletal health in this postpartum period could have important clinical implications for protecting Servicewomen from musculoskeletal injury upon returning to work.

Data from military training show changes in tibial bone morphology and density within 8 to 14 weeks of training in Servicewomen (approximately 0.5 to 2.0%, depending on outcome)^12–14^ and a high risk of bone stress injury^15,16^, indicative of high rates of mechanical loading in military training and employment; increases in whole-body areal bone mineral density (aBMD) are seen over similar time frames^17^. Military training and employment also predispose Servicewomen to tendon injuries^18,19^. Data from 14 weeks military training show no change in patellar tendon cross-sectional area but an increase in damaged fibres in men^20^, which could be a risk factor for subsequent injury^21^. Pregnancy decreases whole-body, lumbar spine, and femoral neck aBMD by approximately 2 to 3% from pre-conception to 1 to 6 weeks postpartum^22–25^, with losses of up to 10% aBMD (site dependent) with 6 months breastfeeding^1,26,27^; there are limited data on how tibial bone structure changes during the postpartum period. Pregnancy can also increase tendon elasticity^2^. Appropriate exercise interventions may protect or help support recovery of these musculoskeletal adaptations in pregnancy. Weight-bearing exercise and resistance training has been proposed as a method to mitigate postpartum bone loss^1^ and protect from stress fractures^28^ and tendon injuries^20^, but any training intervention in service personnel must improve aerobic capacity and strength to support military performance^29^. The proposed optimal training strategy to prepare women for physically demanding military activities include combined high-intensity interval training and heavy upper and lower-body resistance training^30,31^. High-intensity weight bearing interval training and/or resistance training can improve military occupational performance^32^ and elicit positive adaptations to bone^33–35^ and tendon^36^ in women, but there are limited data on the effect of this type of training on bone and tendon adaptations in postpartum women.

This manuscript reports the secondary outcomes from a non-randomised controlled trial examining the effect of an 18-week rehabilitation and endurance and resistance training program in postpartum Servicewomen. Primary outcomes were physical performance and are reported elsewhere^37^. This manuscript reports findings from exploratory analyses of the effect of the intervention on aBMD, tibial macrostructure, and patella tendon morphology and biomechanical properties, and the effect of the postpartum period on these outcomes.

## Methods

### Participants

Servicewomen aged ≥ 18 years in the antenatal and early postpartum (< 6 weeks postpartum) periods were invited to participate by midwives and through advertisements at targeted military sites from July 2019 to September 2022. Participants were eligible if they had completed a 6-week postpartum checkup with their general practitioner and were cleared to exercise. Exclusion criteria were diagnosis of postnatal depression or a mental health condition requiring specialist psychiatric secondary care, or any condition or injury affecting ability to exercise (e.g., Caesarean section). Each participant had the study procedures and risks fully explained verbally and in writing before providing written informed consent. This study was approved by the Ministry of Defence Research Ethics Committee (Ref: 924/MoDREC/18). All experiments were performed in accordance with relevant guidelines and regulations.

### Study design

**Fig. 1:**
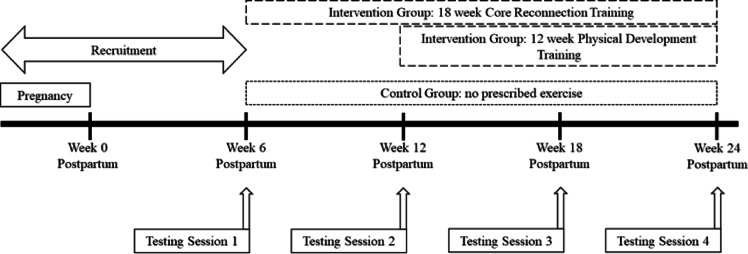
Study overview. Bone outcomes were measured at week 6 and 24 postpartum (Testing Session 1 and 4; week 1 and 18 of the intervention). Tendon outcomes were measured at week 6, 12, and 24 postpartum (Testing Session 1, 2, and 4; week 1, 6, and 18 of the intervention).

This study was a non-randomised controlled trial (ClinicalTrials.gov: NCT04332757, first posted: 24/03/2020) with the protocol previously published^38^. This study is exploratory analyses of secondary musculoskeletal outcomes. There were no public or patient involvement in the design of the trial. Participants were assigned to either a control (Control) or training intervention (Intervention) based on their military unit location. This design was chosen to geographically separate groups to reduce the likelihood of the intervention being shared. The Intervention sites were selected based on their proximity to the study training facilities, with the training completed at Army Headquarters, Andover, UK. The Control group received standard postpartum care with no formal intervention, whereas the Intervention group received standard postpartum care plus an 18-week phased rehabilitation and endurance and resistance training program between 6 and 24 weeks postpartum (Fig. 1). Whole-body aBMD was measured by dual energy X-ray absorptiometry (DXA) and tibial volumetric bone mineral density (vBMD), geometry, and microarchitecture were measured by high-resolution peripheral quantitative computed tomography (HRpQCT) at week 1 and 18 of the intervention (6 and 24 weeks postpartum). Patella tendon morphology and biomechanical properties during isometric contractions were measured by ultrasound at week 1, 6, and 18 of the intervention (6, 12, and 24 weeks postpartum). All testing procedures were completed at the Army Human Performance laboratories, Royal Military Academy, Sandhurst, UK.

### Training intervention

The Control group were not prescribed specific exercises but were not prevented from exercising. The Control group received standard postpartum care, consisting of a 6-week postpartum check. The Intervention group received standard postpartum care plus a phased 18-week combined rehabilitation and endurance and resistance training programme. Rehabilitation included exercises targeting pelvic floor function and core strength from week 6 to 24 postpartum. Training consisted of combined high-intensity interval training and resistance training from weeks 12 to 24 postpartum (see^38^ for full exercise list). The training was targeted to improve physical performance on military relevant tasks^32^. Participants in both groups documented exercise outside of the study using exercise logs.

Following baseline measurements at week 6 postpartum, the Intervention group began an 18-week home-based Core Reconnection programme from weeks 6 to 24 postpartum. Participants completed three ~ 30-minute sessions per week, each consisting of six progressively challenging exercises. Exercises were performed for 3 to 4 sets of 8 to 12 repetitions for each exercise, with modifications based upon individual ability and symptoms. At 12 weeks postpartum, the Intervention group began a supervised 12-week combined high-intensity interval training and resistance training programme^32^. Participants attended three, 90-minute sessions per week. The 12-week programme was divided into three, 4-week blocks, with the first three weeks of each block for progressive training and the final week of each block for ‘de-loading’ and assessing 1-repetition maximums (1RM). Participants completed a 1–5 RM test to determine 1RM, which was used to determine the level of resistance during each block. The target intensity was ≥75% 1RM eliciting between 3 and 12 repetitions, depending on training block and day. Each resistance training session began with a warm-up followed by variations of five exercises: an upper body pull, an upper body push, a lower body pull, a lower body push, and a core exercise. Resistance training exercises included pull-ups, rows, chest presses, deadlifts, and squats. Each of the three weekly training days had a specific focus (e.g., Day 1 = power; Day 2 = strength; Day 3 = stability). High-intensity interval training exercises were completed in the same session as resistance training. High-intensity interval training included upper and lower body exercises, and activities such as running, cycling, jumps and lunges, step ups, and loaded resistance activities (e.g., loaded fast walks, kettle bell exercises). Heart rate (Polar H7, *v*3.4.4) and rating of perceived exertion (0 to 10) were measured during each session to ensure participants were exercising at the prescribed intensity and to progress intensity. The target heart rate was 65 to 95% of estimated maximum heart rate (220 – age), depending on session. Compliance was monitored verbally and through exercise logs. Participants who missed > 25% of rehabilitation sessions over two consecutive weeks, or who failed to attend at least two of the three weekly physical training sessions over the same period, were encouraged to increase participation the following week.

### Areal bone mineral density

Whole-body aBMD was assessed using DXA (Lunar iDXA, GE Healthcare, UK) with participants wearing shorts and a T-shirt. A quality control check scan was performed before each scan using a calibration block with a known density of hydroxyapatite, according to manufacturer instructions. Regional analysis of aBMD for the arms, legs, trunk, pelvis, and spine were derived from the whole-body scan. The coefficient of variation (CV) and least significant change (LSC) for this DXA for whole-body aBMD is 0.5% CV and 1.5% LSC with regional sites for aBMD ≤ 1.5% CV and ≤ 4.2% LSC^13^.

## Tibial volumetric bone mineral density, geometry, and microarchitecture

A three-dimensional HRpQCT scanner (XtremeCT II, Scanco Medical AG, Switzerland) was used to assess vBMD, geometry, and microarchitecture of the tibia in the non-dominant leg. A three-dimensional representation of 10.2 cm of the right tibia in the axial direction, at both the metaphysis (4% site) and diaphysis (30% site), were obtained from 168 CT slices with an isotropic voxel size of 61 μm. Tibial length was measured before the first scan, taken as the distance between the medial malleolus and the tibial proximal endplate. The leg of each participant was fitted into a carbon fibre shell and immobilised within the gantry of the scanner for the duration of the scan. A reference line was placed at the tibial distal endplate, with the first CT slice taken at 4% and 30% of the tibia length from the reference line. For follow-up measurements at the 4% site, automated 2D registration was used and participants with < 75% common region were excluded^39^. The automated matching algorithms were disabled for analysis at the 30% site^12,13,40^. Daily quality control scans were performed using the manufacturer-issued phantom that contained rods of hydroxyapatite (HA). The quality of each HRpQCT scan was reviewed by a single operator and any scans judged to be of poor quality, as per the manufacturer visual grading of image quality, were excluded from the analyses. The standard evaluation procedure provided by the manufacturer was used to derive: total vBMD (mg HA∙cm^3^), trabecular vBMD (mg HA∙cm^3^), cortical vBMD (mg HA∙cm^3^), trabecular area (mm^2^), trabecular bone volume (%), cortical area (mm^2^), cortical thickness (mm), trabecular thickness (µm), trabecular number (1∙mm), trabecular separation (µm), and cortical porosity (%). Micro-finite element analysis was performed to calculate stiffness [kN∙mm] and failure load [kN] under uniaxial compression. All evaluations were performed by a single investigator to ensure consistency of periosteal and endosteal contouring. The coefficient of variations (CV) and least significant changes (LSC) for this HRpQCT at the 4% and 30% sites are ≤ 0.9% CV and ≤ 2.4% LSC for vBMD outcomes, ≤ 1.3% CV and ≤ 3.3% LSC for geometry, ≤ 2.1% CV and ≤ 5.8% LSC for trabecular microarchitecture, ≤ 7.8% CV and ≤ 21.9% LSC for cortical porosity, and ≤ 3.2% CV and ≤ 9.0% LSC for stiffness and failure load^13^.

### Patella tendon elongation

Patella tendon elongation of the dominant leg was assessed using real-time B-mode ultrasonography (MyLab Omega, Esoate) using a linear 4 to 15 Hz probe and acoustic gel during a 5 s ramped maximal isometric knee extension contraction performed at a 90° knee angle on an isokinetic dynamometer (Biodex Isokinetic Dynamometer, System 4). The dominant leg was strapped at the ankle to the knee extension and flexion attachment. Straps were tightly positioned at the shoulder and hip and across the thigh of the measured leg. Isometric ramped contractions were performed over 5 s with participants gradually increasing force against the dynamometer, aiming to reach maximum force within 5 s. Each participant performed four contractions before completing three trial attempts to ensure reproducibility^41^. Visual force feedback was provided from the isokinetic dynamometer. An ecoabsorptive external marker was fixed to the skin using surgical tape to mark the patella tendon. The ultrasound transducer was positioned over the patella tendon and the external marker. Tendon displacement was measured as the distance between the line cast by the external marker and the patella apex. Trials were not analysed if the line cast by the external marker moved on the ultrasound image. A calibrated goniometer was attached to the lateral side of the tested knee to prevent overestimation of tendon elongation due to tibial translation.

### Surface electromyography

Surface electromyography (EMG) was used to measure muscle activity (DataLOG system, MWX8, Biometrics) from the long head of the m. biceps femoris. An EMG sensor (SX230, Biometrics) with 20-mm contact sensor spacing was applied at a site corresponding to the distal one-third of the length of the muscle^41^. The area was shaved and cleaned with alcohol before applying the electrode. The location of the electrode was traced onto an acetate sheet to ensure accurate placement for subsequent tests. The raw EMG signal was pre-amplified and filtered using high- and low-pass cutoff filters set at 10 and 500 Hz (Biometrics Analysis Software, Biomertrics). The root-mean-square EMG activity of the biceps femoris was measured during each isometric ramped contraction to assess the antagonistic coactivation level of the knee flexors. A maximal knee flexion isometric contraction was performed to determine biceps femoris maximal activation when acting as an agonist. The maximal activation of the bicep femoris was measured over a 50 ms window at the point of the maximum torque. The antagonistic torque of the knee flexors during a knee extension contraction was calculated assuming a linear relationship between EMG and the torque^42^ to calculate true knee extensor torque.

### Patella tendon morphology

Resting patella tendon length and cross-sectional area were measured using real-time B mode ultrasonography (MyLab Omega, Esoate) using a linear 4 to 15 Hz probe and acoustic gel at 90° knee angle. The distance between the apex of the patella and the tibial tuberosity, recorded using sagittal ultrasound images, were taken as resting patella tendon length. Patella tendon cross-sectional area was measured using the ultrasound probe placed in the transverse plane, and images captured at 25%, 50%, and 75% of tendon length. Images were analysed using manual boundary demarcation in ImageJ (*v*1.50c; National Institute of Health) with the mean of three images taken at each site; the mean was used for calculating tendon stress.

### Patella tendon stiffness and young’s modulus

Patella tendon force was calculated by dividing the true knee extensor torque by the estimated patella tendon moment arm^43^. Patella tendon stress was calculated by dividing the tendon force by the tendon cross-section area. Tendon strain was calculated as the ratio (%) of tendon displacement to the initial resting tendon length. Force-elongation data was fitted with a second-order polynomial curve, allowing the assessment of patella tendon stiffness. The Young’s modulus was calculated as tendon stiffness multiplied by the ratio of tendon length to tendon cross-sectional area. Patella tendon force, stress, strain, and Young’s modulus were assessed at 10% MVC increments during the 5 s ramped MVC contraction.

### Statistical analyses

All data were analysed using the R programming language (*v.*4.4.1). The outcomes presented in this manuscript are secondary outcomes and so no sample size calculation was performed for the outcomes reported here^38^. The sample size and available cohort were originally determined based on power calculations for physical performance outcomes^38^; therefore this analysis is secondary and reflects the existing sample rather than a prospectively calculated sample size. Linear mixed effect models with restricted maximum likelihood estimation were used to examine changes in aBMD, tibial structure, and patella tendon properties between Control and Intervention. A repeated measures approach was taken so the effect of the postpartum period on bone and tendon outcomes could be explored. Group (Control vs. Intervention), time (week 6 vs. week 12 [patella tendon properties only] vs. week 24), and their interaction were included as fixed effects. Time was included as a factor rather than numeric variable. Random intercepts were assigned to each participant to account for within participant correlation for repeated measures. Random slopes were not added to the model to reduce model complexity with the small sample size. The significance of the fixed effects was determined with Satterthwaite degrees of freedom (*anova* function, *lmerTest* package *v*.3.1.3). In the event of a significant group × time interaction, pairwise uncorrected comparisons were used to identify differences between time points or groups (*emmeans* packag*e v*.1.10.4). Pooled data were used for main effects of time or group when there was no significant interaction, and each group was analysed independently when there was a significant interaction. Normality of the residuals were checked visually by plotting the residuals against the fitted values and from Q-Q plots. Box-Cox transformations (*MASS* packag*e v*.7.3–60.2) and log transformations were explored when residuals were heteroscedastic or non-normal. Trabecular area, trabecular thickness, cortical area at the 4% and 30% sites, cortical vBMD, perimeter, porosity, and thickness at the 30% site, stiffness at the 30% site, and failure load at the 4% and 30% site had residuals with long-tailed distributions. Box-Cox or logarithmic transformations made no substantial difference to the model fit or interpretation and so models on the original untransformed data are presented. Effect sizes are presented as partial eta-squared (η_p_^2^) for main and interaction effects and paired Hedges’ *g* for within-group paired comparisons (*effectsize* package *v*.0.6.0.1). Figures were drawn in the *ggplot2* package (*v.*3.4.2). Significance was accepted as *p* ≤ 0.05.

## Results

### Participants

**Fig. 2:**
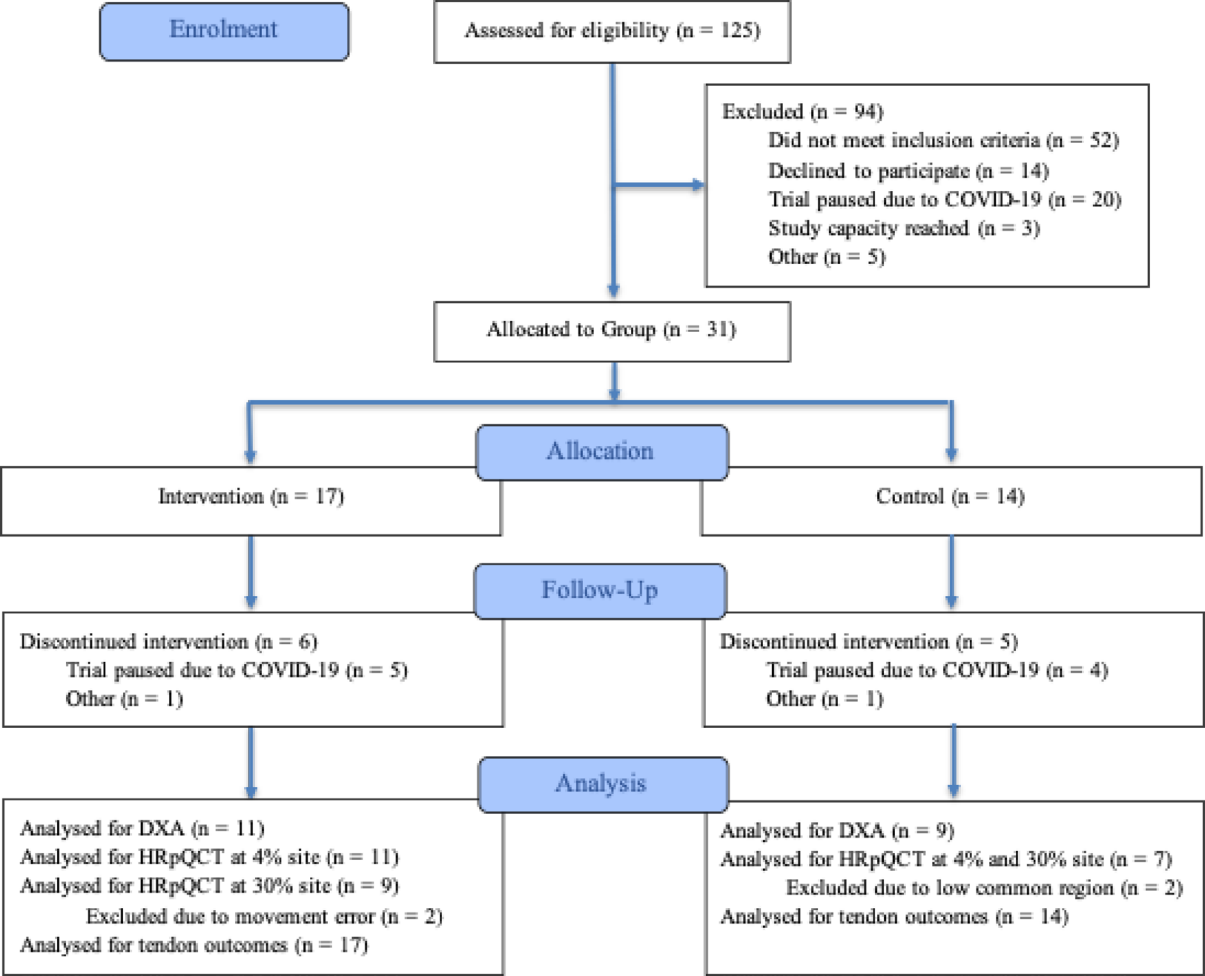
Participant flow through the study.DXA, dual energy X-ray absorptiometry.HRpQCT, high-resolution peripheral quantitative computed tomography.

A total of 31 participants were consented (Intervention, *n* = 17; Control, *n* = 14) (Table 1). A total of 11 participants were lost to follow-up (Fig. 2) with 20 participants having week 1 and 18 DXA and HRpQCT scans (Intervention group, *n* = 11; Control group, *n* = 9). No participant incurred an injury as part of the training. The Intervention group completed on average 52 out of 54 of the rehabilitation sessions and 26 out of the 27 endurance and resistance training sessions. The training intensity (RPE and heart) are presented in Supplementary Tables 1, 2, and 3. Two participants from the Control group were excluded from HRpQCT analyses for having common regions < 75% and two participants from the Intervention group were excluded for 30% site scans only due to movement error. The common regions for 4% site scans were (mean ± SD [minimum]): Control = 95 ± 4% [88%]; Intervention = 96 ± 4% [87%]. All participants were included in analysis of tendon properties to allow use of partial follow-up data at week 6 of the intervention.

**Table 1:** Participant demographics. Data are mean ± SD, mean, or %.

Age (years)	Control (*n* = 14)	Intervention (*n* = 17)
	35 ± 4	33 ± 4
Height (m)	1.63 ± 0.05	1.68 ± 0.05
Body Mass (kg)	67.9 ± 9.6	72.9 ± 9.5
Breastfeeding (%)		
* Week 1*	86	82
* Week 6*	56	82
* Week 18*	44	64
Resumption of menses at week 18 (%)	56	55
Hormonal contraceptive use at week 18 (%)	33	55
Number of the 54 rehabilitation sessions completed (mean)	N/A	52
Number of the 27 training sessions completed (mean)	N/A	26
Number of total exercise bouts (intervention and personal)		
* Week 1 to 6*	35 ± 15	31 ± 15
* Week 7 to 12*	32 ± 14	46 ± 16
* Week 13 to 18*	32 ± 19	48 ± 18

### Areal bone mineral density

Table 2 shows the whole-body and regional aBMD data. There was no group × time interaction (*p* ≥ 0.100, η_p_^2^ ≤ 0.143) or main effect of group (*p* ≥ 0.084, η_p_^2^ ≤ 0.156) for any measure of aBMD. There were main effects of time for aBMD of the trunk and spine (*p* ≤ 0.021, η_p_^2^ ≤ 0.262) with both measures decreasing between week 1 and 18 when the level of group was collapsed.

### Tibial volumetric bone mineral density, geometry, microarchitecture, and estimated mechanical strength

Table 3 shows the tibial vBMD, geometry, microarchitecture, and estimated mechanical strength outcome data. There were no group × time interactions for any tibial density, geometry, microarchitecture, or estimated strength outcome (*p* ≥ 0.151, η_p_^2^ ≤ 0.124). There were no main effects of time for any outcome (*p* ≥ 0.057, η_p_^2^ ≤ 0.209) but there were main effects of group for cortical perimeter, stiffness, and failure load at the 30% site (*p* ≤ 0.049, η_p_^2^ ≤ 0.274), which were all larger in Intervention compared with Control group.

**Table 2:** Whole-body and regional areal bone mineral density in postpartum Servicewomen before and after 18 weeks of standard care (Control, *n* = 9) or standard care plus rehabilitation and endurance and strength training (Intervention, *n* = 11).

	Control (*n* = 9)				Intervention (*n* = 11)				Group × time
	Week 1	Week 18	Mean difference [95% CI]	Hedges’ g [95% CI]	Week 1	Week 18	Mean difference [95% CI]	Hedges’ g [95% CI]	η_*p*_^2^ [95% CI]
Whole-body aBMD (g∙cm^− 3^)	1.21 ± 0.06	1.20 ± 0.08	−0.01 [−0.03, 0.02]	−0.21 [−0.80, 0.40]	1.26 ± 0.08	1.25 ± 0.07	−0.01 [−0.03, 0.01]	−0.43 [−1.00, 0.15]	0.01 [0.00, 1.00]
Arms aBMD (g∙cm^− 3^)	0.78 ± 0.05	0.76 ± 0.06	−0.01 [−0.04, 0.01]	−0.34 [−0.94, 0.29]	0.80 ± 0.09	0.76 ± 0.06	−0.03 [−0.07, 0.01]	−0.47 [−1.05, 0.12]	0.04 [0.00, 1.00]
Legs aBMD (g∙cm^− 3^)	1.26 ± 0.07	1.26 ± 0.07	0.00 [−0.02, 0.03]	0.13 [−0.46, 0.72]	1.32 ± 0.10	1.33 ± 0.09	0.01 [−0.01, 0.04]	0.36 [−0.22, 0.92]	0.02 [0.00, 1.00]
Trunk aBMD (g∙cm^− 3^)	1.05 ± 0.07	1.04 ± 0.10^a^	−0.01 [−0.04, 0.02]	−0.26 [−0.86, 0.35]	1.07 ± 0.08	1.05 ± 0.08^a^	−0.02 [−0.03, 0.01]	−1.50 [−2.34, −0.64]	0.04 [0.00, 1.00]
Pelvis aBMD (g∙cm^− 3^)	1.11 ± 0.03	1.10 ± 0.06	−0.01 [−0.05, 0.03]	−0.14 [−0.73, 0.46]	1.14 ± 0.08	1.13 ± 0.08	−0.01 [−0.03, 0.00]	−0.53 [−1.11, 0.07]	0.01 [0.00, 1.00]
Spine aBMD (g∙cm^− 3^)	1.13 ± 0.07	1.12 ± 0.09^a^	−0.01 [−0.04, 0.03]	−0.15 [−0.74, 0.45]	1.20 ± 0.11	1.16 ± 0.09^a^	−0.04 [−0.06, −0.01]	−0.94 [−1.61, −0.25]	0.14 [0.00, 1.00]

aBMD, areal bone mineral density; vBMD, volumetric bone mineral density.^a^p < 0.05 vs week 1 (main effect of time, both groups pooled).

**Table 3:** Tibial volumetric bone mineral density, geometry, microarchitecture, and estimated mechanical strength in postpartum Servicewomen before and after 18 weeks of standard care (Control, *n* = 7) or standard care plus rehabilitation and endurance and resistance training (Intervention, *n* = 11).

	Control (*n* = 7)				Intervention (*n* = 11)				Group × time
	Week 1	Week 18	Mean difference [95% CI]	Hedges’ g [95% CI]	Week 1	Week 18	Mean difference [95% CI]	Hedges’ g [95% CI]	η_*p*_^2^ [95% CI]
Tibial metaphysis (4% Site)									
Total vBMD (mgHA∙cm^− 3^)	250 ± 32	248 ± 33	−3 [−10, 4]	−0.33 [−0.99, 0.35]	251 ± 27	249 ± 26	−2 [−4, 0]	−0.57 [−1.15, 0.04]	0.01 [0.00, 1.00]
Trabecular vBMD (mgHA∙cm^− 3^)	205 ± 21	201 ± 23	−4 [−12, 4]	−0.39 [−1.05, 0.30]	204 ± 20	203 ± 18	−2 [0, 4]	−0.57 [−1.15, 0.04]	0.03 [0.00, 1.00]
Trabecular Area (mm^− 2^)	941 ± 103	941 ± 100	−0 [−7, 7]	−0.03 [−0.67, 0.62]	958 ± 133	954 ± 126	−4 [−16, 8]	−0.20 [−0.75, 0.36]	0.02 [0.00, 1.00]
Trabecular volume (%)	28.7 ± 3.8	28.3 ± 4.0	−0.4 [−1.6, 0.8]	−0.29 [−0.94, 0.39]	28.7 ± 2.9	28.5 ± 2.7	−0.3 [−0.8, 0.2]	−0.34 [−0.90, 0.23]	0.01 [0.00, 1.00]
Trabecular number (1∙mm)	1.75 ± 0.16	1.73 ± 0.13	−0.02 [−0.10, 0.07]	−0.15 [−0.79, 0.51]	1.78 ± 0.17	1.76 ± 0.17	−0.02 [−0.09, 0.04]	−0.22 [−0.77, 0.34]	0.00 [0.00, 1.00]
Trabecular thickness (µm)	258 ± 28	245 ± 14	−13 [−40, 14]	−0.39 [−1.05, 0.30]	250 ± 17	245 ± 17	−5 [−7, −2]	−1.17 [−1.90, −0.41]	0.05 [0.00, 1.00]
Trabecular separation (µm)	534 ± 45	536 ± 39	2 [21, 26]	0.08 [−0.57, 0.72]	523 ± 54	528 ± 51	6 [−10, 21]	0.22 [−0.34, 0.77]	0.00 [0.00, 1.00]
Cortical vBMD (mgHA∙cm^− 3^)	760 ± 70	763 ± 62	3 [−8, 14]	0.23 [−0.43, 0.88]	744 ± 67	749 ± 63	5 [−7, 18]	0.27 [−0.29, 0.82]	0.01 [0.00, 1.00]
Cortical Area (mm^− 2^)	83 ± 21	85 ± 17	2 [−3, 7]	0.32 [−0.36, 0.98]	88 ± 14	87 ± 13	−1 [−3, 1]	−0.26 [−0.81, 0.30]	0.12 [0.00, 1.00]
Cortical Thickness (mm^− 1^)	0.73 ± 0.21	0.76 ± 0.16	0.03 [−0.03, 0.09]	0.35 [−0.34, 1.01]	0.79 ± 0.16	0.78 ± 0.14	−0.01 [−0.03, 0.01]	−0.25 [−0.36, 0.84]	0.12 [0.00, 1.00]
Cortical Porosity (%)	1.13 ± 0.70	1.17 ± 0.66	0.04 [−0.07, 0.16]	0.29 [−0.38, 0.94]	1.27 ± 0.42	1.27 ± 0.41	0.00 [−0.18, 0.18]	0.00 [0.00, 0.00]	0.01 [0.00, 1.00]
Stiffness (kN∙mm^− 1^)	164 ± 55	168 ± 50	3 [−9, 16]	0.23 [−0.44, 0.87]	165 ± 28	169 ± 28	4 [−9, 17]	0.17 [−0.38, 0.72]	0.00 [0.00, 1.00]
Failure Load (kN)	8.92 ± 2.83	9.08 ± 2.62	0.16 [−0.40, 0.72]	0.22 [−0.44, 0.87]	9.95 ± 3.06	9.24 ± 1.41	−0.71 [−2.97, 1.55]	−0.20 [−0.74, 0.36]	0.03 [0.00, 1.00]
Tibial diaphysis (30% Site)*									
Cortical vBMD (mgHA∙cm^− 3^)	1,060 ± 24	1,063 ± 22	3 [−5, 11]	0.27 [−0.40, 0.92]	1,038 ± 28	1,039 ± 28	2 [0, 4]	0.64 [−0.04, 1.28]	0.00 [0.00, 1.00]
Cortical area (mm^− 2^)	245 ± 26	245 ± 26	0 [−1, 2]	0.12 [−0.53, 0.76]	272 ± 29	276 ± 29	4 [−5, 12]	0.28 [−0.33, 0.88]	0.04 [0.00, 1.00]
Cortical thickness (mm^− 1^)	5.80 ± 0.61	5.81 ± 0.63	0.01 [−0.03, 0.05]	0.21 [−0.45, 0.86]	6.19 ± 0.62	6.28 ± 0.49	0.10 [−0.18, 0.37]	0.25 [−0.36, 0.84]	0.03 [0.00, 1.00]
Cortical perimeter (mm^− 1^)	71.6 ± 4.0	71.6 ± 4.0	0.1 [−0.3. 0.4]	0.17 [−0.49, 0.81]	76.3 ± 4.2^a^	76.7 ± 4.5^a^	0.4 [−0.4, 1.1]	0.34 [−0.28, 0.94]	0.04 [0.00, 1.00]
Cortical porosity (%)	0.60 ± 0.41	0.63 ± 0.45	0.03 [−0.02, 0.07]	0.51 [−0.21, 1.19]	0.61 ± 0.41	0.57 ± 0.45	−0.04 [−0.24, 0.15]	−0.16 [−0.75, 0.44]	0.04 [0.00, 1.00]
Stiffness (kN∙mm^− 1^)	256 ± 27	257 ± 28	1 [0, 3]	0.70 [−0.07, 1.43]	288 ± 32^a^	291 ± 31^a^	3 [−6, 12]	0.23 [−0.38, 0.82]	0.01 [0.00, 1.00]
Failure load (kN)	14.42 ± 1.55	14.59 ± 1.62	0.17 [−0.06, 0.40]	0.60 [−0.14, 1.30]	16.27 ± 1.93^a^	16.40 ± 1.66^a^	0.13 [−0.42, 0.68]	0.16 [−0.44, 0.75]	0.00 [0.00, 1.00]

*n = 9 for Intervention.^a^p < 0.05 vs Control (main effect of group, both time-points pooled).vBMD, volumetric bone mineral density.

### Patella tendon properties

Table 4 shows tendon morphology and maximal biomechanical properties with mean differences and effect sizes shown in Table 5. There were no group × time interactions for any measure of tendon morphology or maximal biomechanical properties (*p* ≥ 0.185, η_p_^2^ ≤ 0.078). There were main effects of time for knee extension maximum voluntary contraction force, maximum tendon force, and maximum tendon stress (*p* ≤ 0.044, η_p_^2^ ≥ 0.159). Knee extension maximum voluntary contraction force (*p* = 0.023), maximum tendon force (*p* = 0.034), and maximum tendon stress (*p* = 0.018) increased from week 1 to 18 when the level of group was collapsed. There were main effects of group for tendon length and maximal tendon strain (*p* ≤ 0.031, η_p_^2^ ≤ 0.265); tendon length was larger in the Intervention compared with the Control group whereas tendon strain was larger in the Control compared with the Intervention group.

**Fig. 3:**
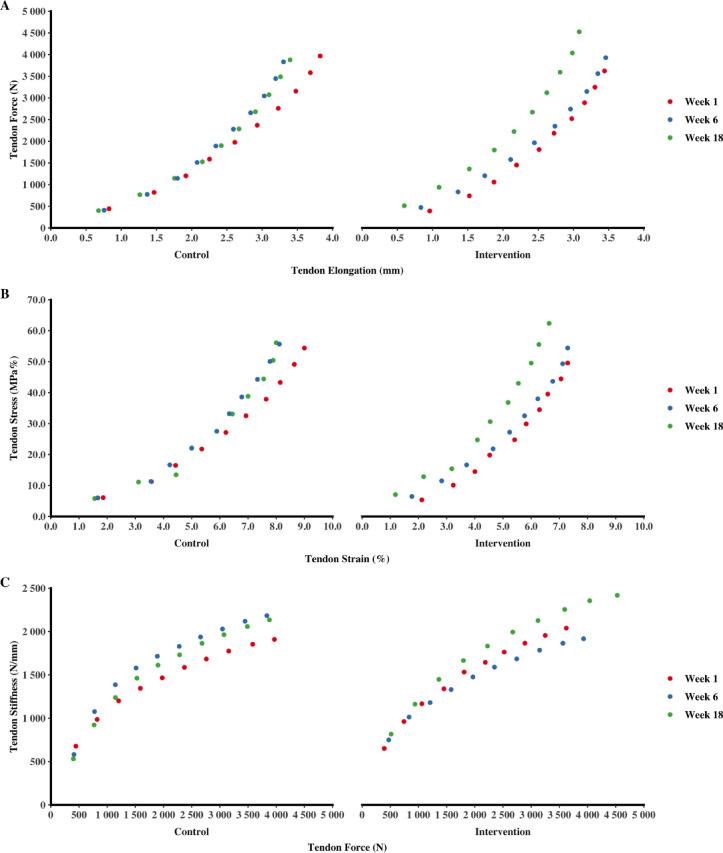
Patella tendon force-elongation (**A**), stress-strain (**B**), and force-stiffness (**C**) curves in postpartum Servicewomen before (week 1), during (week 6), and after (week 18) 18 weeks of standard care (Control) or standard care plus rehabilitation and endurance and resistance training (Intervention). Data points are mean for the group at that time-point.

Patella tendon force-elongation, stress-strain, and force-stiffness are shown in Fig. 3. There was a group × time interaction for tendon force at 10% MVC (*p* = 0.035, η_p_^2^ = 0.139) but no group × time interaction for tendon elongation, force, stiffness, stress, or Young’s modulus at any contraction intensity (*p* ≥ 0.069, η_p_^2^ ≤ 0.112). Tendon force at 10% MVC did not change over time in the Control group (*p* ≥ 0.743) but increased from week 1 to 18 in the Intervention group (*p* = 0.013). Tendon force at 10% MVC was not different between the Control and Intervention groups at any time-point (*p* ≥ 0.096). There were main effects of time for elongation at 10% and 20% MVC, tendon stress at 40%, 70%, 80%, and 90% MVC, and tendon strain at 10% and 20% MVC (*p* ≤ 0.048, η_p_^2^ ≥ 0.221). Tendon elongation at 10% and 20% MVC decreased from week 1 to 18 (*p* ≤ 0.013) when the level of group was collapsed. Tendon stress at 40%, 70%, 80%, and 90% MVC increased from week 1 to 18 (*p* ≤ 0.039) when the level of group was collapsed. Tendon strain at 10% and 20% MVC decreased from week 1 to 18 (*p* ≤ 0.006) when the level of group was collapsed. There was a main effect of group for tendon strain at 90% MVC (*p* = 0.037, η_p_^2^ = 0.153) with tendon strain larger in the Control than the Intervention group.

**Table 4:** Patella tendon biomechanical properties in postpartum Servicewomen before, during, and after 18 weeks of standard care (Control, *n* = 14) or standard care plus rehabilitation and endurance and resistance training (Intervention, *n* = 17).

	Control (*n* = 14)			Intervention (*n* = 17)			Group ×time
	Week 1	Week 6*	Week 18*	Week 1	Week 6	Week 18^#^	η_*p*_^2^ [95% CI]
Tendon cross-sectional area (mm^2^)	73.2 ± 8.6	70.2 ± 9.0	70.1 ± 8.9	73.7 ± 11.4	74.6 ± 11.5	74.2 ± 10.0	0.05 [0.00, 1.00]
Tendon length (mm)	42.9 ± 3.9	41.6 ± 3.8	41.6 ± 3.5	47.6 ± 4.5^b^	47.4 ± 4.7^b^	47.3 ± 4.0^b^	0.01 [0.00, 1.00]
Knee extensor MVC force (N)	143 ± 50	138 ± 29	141 ± 19^a^	127 ± 52	139 ± 43	159 ± 39^a^	0.08 [0.00, 1.00]
Maximum tendon stiffness (N∙mm^− 1^)	1,968 ± 1,049	2,184 ± 867	2,203 ± 685	2,040 ± 1,041	1,917 ± 812	2,418 ± 628	0.05 [0.00, 1.00]
Maximum tendon force (N)	3,968 ± 1,320	3,834 ± 698	3,879 ± 456^a^	3,623 ± 1,496	3,929 ± 1,138	4,528 ± 934^a^	0.08 [0.00, 1.00]
Maximum tendon elongation (mm)	3.82 ± 1.17	3.30 ± 0.36	3.40 ± 0.47	3.44 ± 0.81	3.46 ± 0.95	3.08 ± 0.51	0.03 [0.00, 1.00]
Maximum young’s modulus (GPa)	1.17 ± 0.64	1.33 ± 0.64	1.27 ± 0.44	1.31 ± 0.67	1.27 ± 0.66	1.56 ± 0.45	0.04 [0.00, 1.00]
Maximum tendon stress (MPa)	54.4 ± 16.7	55.7 ± 13.6	56.1 ± 9.4^a^	49.6 ± 21.3	54.4 ± 19.5	62.4 ± 16.6^a^	0.07 [0.01, 1.00]
Maximum tendon strain (%)	9.00 ± 2.75	8.11 ± 1.17	8.00 ± 1.41	7.29 ± 1.69^b^	7.29 ± 2.02b	6.64 ± 1.21^b^	0.00 [0.00, 1.00]

*n = 9.^#^n = 11.^a^p < 0.05 vs Pre-Training (main effect of time, both groups pooled).^b^p < 0.05 vs Control (main effect of group, all time-points pooled).MVC, maximal voluntary contraction.

**Table 5:** Mean differences and effect sizes for changes in patella tendon biomechanical properties in postpartum Servicewomen before, during, and after 18 weeks of standard care (Control, *n* = 9) or standard care plus rehabilitation and endurance and resistance training (Intervention, *n* = 11).

	Week 1 vs. week 6		Week 1 vs. week 18	
	Mean difference [95% CI]	Hedges’ g [95% CI]	Mean difference [95% CI]	Hedges’ g [95% CI]
Control (*n* = 9)				
Tendon cross-sectional area (mm^2^)	−0.2 [−0.8, 0.5]	−0.18 [−0.77, 0.42]	−0.2 [−1.0, 0.6]	−0.17 [−0.76, 0.43]
Tendon length (mm)	−0.2 [−1.5, 1.0]	−0.14 [−0.73, 0.46]	−0.3 [−1.8, 1.2]	−0.15 [−0.74, 0.45]
Knee extensor MVC force (N)	8 [−6, 22]	0.39 [−0.24, 1.00]	10 [−9, 30]	0.36 [−0.26, 0.97]
Maximum tendon stiffness (N∙mm^− 1^)	217 [−134, 568]	0.43 [−0.21, 1.04]	137 [−443, 298]	0.18 [−0.45, 0.79]
Maximum tendon force (N)	187 [−235, 609]	0.31 [−0.31, 0.91]	233 [−298, 763]	0.30 [−0.31, 0.90]
Maximum tendon elongation (mm)	−0.03 [−0.34, 0.28]	−0.06 [−0.65, 0.53]	0.07 [−0.46, 0.60]	0.09 [−0.51, 0.68]
Maximum young’s modulus (GPa)	0.15 [−0.10, 0.40]	0.42 [−0.21, 1.03]	0.09 [−0.26, 0.43]	0.18 [−0.43, 0.77]
Maximum tendon stress (MPa)	3.1 [−2.8, 9.0]	0.37 [−0.26, 0.97]	3.6 [−4.5, 11.6]	0.31 [−0.31, 0.91]
Maximum tendon strain (%)	0.11 [−0.70, 0.92]	0.10 [−0.50, 0.68]	0.00 [−1.27, 1.27]	0.00 [−0.59, 0.59]
Intervention (*n* = 11)				
Tendon cross-sectional area (mm^2^)	0.8 [−0.3, 2.0]	0.34 [−0.13, 0.81]	0.6 [−1.2, 2.3]	0.19 [−0.37, 0.73]
Tendon length (mm)	−0.2 [−0.8, 4.3]	−0.16 [−0.61, 0.30]	0.0 [−0.7, 0.7]	0.02 [−0.53, 0.56]
Knee extensor MVC force (N)	12 [−4, 27]	0.36 [−0.11, 0.83]	29 [−5, 63]	0.53 [−0.07, 1.11]
Maximum tendon stiffness (N∙mm^− 1^)	−123 [−427, 181]	−0.20 [−0.65, 0.26]	293 [−473, 1060]	0.24 [−0.32, 0.79]
Maximum tendon force (N)	306 [−140, 752]	0.34 [−0.14, 0.80]	760 [−117, 1636]	0.54 [−0.07, 1.12]
Maximum tendon elongation (mm)	0.07 [−0.18, 0.21]	0.04 [−0.41, 0.49]	−0.15 [−0.48, 0.19]	−0.27 [−0.82, 0.30]
Maximum young’s modulus (GPa)	−0.04 [−0.23, 0.14]	−0.11 [−0.56, 0.35]	0.21 [−0.27, 0.70]	0.27 [−0.29, 0.82]
Maximum tendon stress (MPa)	4.9 [−1.7, 11.4]	0.36 [−0.11, 0.83]	11.2 [−1.0, 23.3]	0.57 [−0.04, 1.15]
Maximum tendon strain (%)	0.00 [−0.45, 0.45]	0.00 [0.00, 0.00]	−0.27 [−1.07, 0.53]	−0.21 [−0.76, 0.35]

MVC, maximal voluntary contraction.

## Discussion

This study was unable to detect evidence that an 18-weeks of rehabilitation and whole-body endurance and resistance training programme improved whole-body aBMD, tibial bone density or structure, or patella tendon properties in postpartum Servicewomen. Spinal aBMD decreased, measures of tendon force and tendon stress increased, and tendon elongation and strain decreased, from week 1 to week 18 of the study but there was no evidence of changes in other outcomes. The exercise program was safe and well adhered to, providing evidence that endurance and resistance training can be a method to support postpartum Servicewomen returning to full military duty^37^. These data also provide novel insight into skeletal and tendon health in postpartum women and the effects of chronic exercise training.

This study was unable to detect evidence that training improved whole-body or regional aBMD. A total of 16 weeks combined endurance and resistance training in postpartum breastfeeding women mitigated the loss of aBMD in lumbar spine but not losses in the whole-body or hip^44^, and these training benefits persisted 12 months postpartum^45^. Our 12-week endurance and resistance training period was shorter in duration than these 16-week training studies, which may explain why we did not detect evidence of an effect of training. Other evidence shows 16 weeks of resistance training (combined with energy restriction) had no effect on whole-body, lumbar spine, or hip aBMD in postpartum breastfeeding women^46^, and observational studies show no association between physical activity volume in the first 3 to 12 months postpartum and lumbar spine or femur aBMD changes^47,48^. There were few data on exercise training on bone in the postpartum period, and data from this study suggest endurance and resistance training has modest effects on aBMD. We observed decreases in trunk (approximately 1% in the Control group and 2% in the Intervention group) and spine (approximately 1% in the Control group and 3% in the Intervention group) aBMD from week 1 to 18. These changes are close to the limit of LSC and must be interpreted with caution. Pregnancy can decrease whole-body, lumbar spine, and femoral neck aBMD by approximately 2 to 3% from pre-conception to 1 to 6 weeks postpartum^22–25^, but most foetal calcium requirements during pregnancy are met through increased intestinal calcium absorption as a result of increased 1,25 dihydroxyvitamin D production rather than from maternal bone^1,26^. Areal bone mineral density in the postpartum period depends on breastfeeding status. Declines in aBMD are seen for up to 12 months postpartum in those breastfeeding compared with increases or no change in aBMD in those not (or stopping) breastfeeding^22,24,49^. During breastfeeding, the maternal skeleton is resorbed during lactation to provide calcium for milk, with this osteoclastic resorption driven by breast-derived parathyroid hormone related protein and low oestradiol^1,26^. Losses of approximately 5 to 10% aBMDࣧat mainly trabecular sitesࣧare reported with 6 months breastfeeding compared with immediately postpartum, with 0 to 2% losses in whole-body aBMD^1,26,27^; this loss of aBMD fully recovers approximately 6 to 12 months following the completion of weaning^1,50,51^.

This study was unable to detect evidence that training improved tibial density, geometry, microarchitecture, or estimated strength. The training programme in this study was aimed at improving physical performance on military occupational tasks rather than specifically targeting bone or tendon adaptations, and there may have been insufficient loading stimulus to induce mechano-adaptation in tibial bone. However, weight-bearing impact training and resistance training can increase the density and size of the tibia bone in young and older women^34,52^. The women in this study were trained military personnel who had already completed a period of military service and so further adaptations in the tibia with weight-bearing training could be less likely in this population. Finally, the endurance and resistance training programme was performed in the last 12 weeks of this 18-week study, which may not have been long enough to induce changes in tibial bone at the dose prescribed. Additionally, many other studies have designed the resistance training to elicit skeletal health benefits whereas the training in this study was intended to improve military occupational task performance. However, the training likely caused a number of bone modeling and remodeling cellular responses that can take longer than the follow-up time in this study to observe structural or mechanical effects^28,53^. We did not detect evidence of a change in tibial bone density or structure from week 1 to 18 of the study. Pregnancy can decrease trabecular thickness and separation, and cortical vBMD and thickness at the tibial metaphysis^54^, and women with postpartum osteoporosis have smaller tibial bone size and density than age-matched controls^55^. The postpartum period can also result in decreases in tibial metaphyseal trabecular vBMD, volume, and thickness, and cortical vBMD, area, and thickness, with these changes associated with breastfeeding^49^.

This study was unable to detect evidence that training improved patella tendon morphology or maximal biomechanical properties. Previous studies of resistance training in young women show increases in patella tendon cross sectional area^56,57^ and tendon stiffness^56,58^. Whilst we did not observe group differences, the Control group were physically active, which may have induced adaptations in tendon meaning we were not able to detect a training effect in our study; the increases in maximal voluntary contraction force and maximum tendon force irrespective of group support this supposition. High magnitudes of load are most beneficial for tendon adaption (e.g., contraction intensities > 70% 1RM)^36^ and it is also not clear if our training programme used sufficient loads to cause measurable adaptation compared with Controls. During ramped maximal contractions, tendon force at 10% MVC increased from week 1 to 18 in Intervention but not Control, but the large number of statistical comparisons mean this single group difference could be due to Type I error. Similarly, tendon elongation decreased from week 1 to week 18 during ramped maximal contractions (irrespective of group), which could also be a result of Type I error. The curvilinear force-elongation and stress-strain curves appear to have shifted to the left (Fig. 3)ࣧ mainly in the Intervention groupࣧwhich could be consistent with adaptations to lower body resistance training^41,56^. Pregnancy can result in increased tendon elasticity due to increases in oestradiol^2^ but a previous observational study showed no mal-adaptive change in patella tendon mechanical properties during pregnancy or the postpartum period^59^, consistent with our study. The implications of this training programme on reducing tendon injury risk is not clear but tendons are metabolically active structures and their properties can adapt to exercise training^36,41^; abnormal adaptations may, however, lead to tendinopathy, injury, or pain.

The sample size in this study was small, in part, due to disruptions from COVID-19, and we were underpowered to detect small effects of exercise training on tibial bone and patella tendon properties. The HRpQCT and patellar tendon measures produce many outcomes, which increases the chance of Type I error. Our study did not include measures of vitamin D status, PTH, oestradiol, or markers of bone metabolism, which may help explain some of our findings. Our study did not include a lumbar spine, hip, or femur DXA scan, which may have provided greater measurement sensitivity than the whole-body scan. We also did not have a measure of mechanical loading or impact loading. The control group were ‘free living’, meaning that participants could undertake their won exercise during the study period, which precludes comparisons with a no-exercise control. Pre-pregnancy and antenatal training status and exercise history were not assessed, which may have influenced responsiveness to the intervention; however, all participants were Servicewomen in the British Army and so had all completed military basic training, were mandated to complete miliary physical training three times per week, and were required to complete and pass the same annual physical fitness tests before pregnancy.

This study was unable to detect evidence that 18-weeks combined rehabilitation and endurance and resistance training programme improved whole-body aBMD, tibial bone density, structure, or strength, or patella tendon properties in postpartum Servicewomen. Training that specifically targets bone and tendon may be required to cause adaptations in these tissues. Spinal aBMD decreased, consistent with the effects of breastfeeding on bone mass.

## Supplementary Information

Below is the link to the electronic supplementary material.

Supplementary Material 1

## Data Availability

The data that support the findings of this study are available from the corresponding author pending approval from the UK Ministry of Defence.
