# Supplementary material for: Bone and tendon adaptations to 18-weeks rehabilitation and endurance and resistance training in postpartum British Servicewomen: a non-randomised controlled trial
Source: Sci Rep. 2026 May 2;16:20308. doi: 10.1038/s41598-026-51411-3 (PMC13324743; doi:10.1038/s41598-026-51411-3)
Supplement: Supplementary file 1 — Supplementary Material 1 [file 41598_2026_51411_MOESM1_ESM.docx]

|  | Week 1 | Week 2 | Week 3 |
| --- | --- | --- | --- |
| Mesocycle 1 Day 1 | 4 ± 1 | 5 ± 1 | - |
| Mesocycle 1 Day 2 | 7 ± 1 | 6 ± 0 | - |
| Mesocycle 1 Day 3 | 6 ± 1 | 6 ± 1 | - |
| Mesocycle 2 Day 1 | 6 ± 1 | - | - |
| Mesocycle 2 Day 2 | 7 ± 1 | - | - |
| Mesocycle 2 Day 3 | 7 ± 1 | - | - |
| Mesocycle 3 Day 1 | 7 ± 1 | 7 ± 1 | 8 ± 1 |
| Mesocycle 3 Day 2 | 7 ± 1 | 8 ± 1 | 9 ± 1 |
| Mesocycle 3 Day 3 | 7 ± 1 | 7 ± 0 | 8 ± 1 |
| Mesocycle 4 Day 1 | 7 ± 1 | 8 ± 1 | 8 ± 1 |
| Mesocycle 4 Day 2 | 8 ± 1 | 8 ± 1 | 9 ± 1 |
| Mesocycle 4 Day 3 | 8 ± 1 | 8 ± 2 | 8 ± 1 |

**Supplementary Table 1.** Ratings of perceived exertion (10-point scale) for resistance training. Data are mean ± SD.

**Supplementary Table 2.** Ratings of perceived exertion (10-point scale) for high-intensity interval training. Data are mean ± SD.

|  | Day | Week 1 | Week 2 | Week 3 |
| --- | --- | --- | --- | --- |
| Mesocycle 1 and 2 | 1 | 6 ± 1 | 7 ± 1 | 7 ± 1 |
|  | 2 | 7 ± 1 | 7 ± 1 | 7 ± 1 |
|  | 3 | 7 ± 1 | 7 ± 1 | 7 ± 1 |
| Mesocycle 3 | 1 | 8 ± 1 | 8 ± 1 | 8 ± 1 |
|  | 2 | 7 ± 1 | 7 ± 1 | 8 ± 1 |
|  | 3 | 7 ± 1 | 7 ± 1 | 8 ± 1 |
| Mesocycle 4 | 1 | 8 ± 1 | 8 ± 1 | 8 ± 1 |
|  | 2 | 8 ± 1 | 8 ± 1 | 8 ± 1 |
|  | 3 | 8 ± 1 | 8 ± 1 | 8 ± 1 |

**Supplementary Table 3.** Heart rate for high-intensity interval training. Data are mean ± SD.

|  |  | Target Heart Rate (b·min^-1^) | | | Average Heart Rate (b·min^-1^) | | | Peak Heart Rate (b·min^-1^) | | |
| --- | --- | --- | --- | --- | --- | --- | --- | --- | --- | --- |
|  | Day | Week 1 | Week 2 | Week 3 | Week 1 | Week 2 | Week 3 | Week 1 | Week 2 | Week 3 |
| Mesocycle 1 and 2 | 1 | 139 ± 2 | 149 ± 2 | 158 ± 3 | 145 ± 13 | 149 ± 11 | 151 ± 13 | 172 ± 12 | 170 ± 15 | 173 ± 13 |
|  | 2 | 144 ± 3 | 153 ± 3 | 166 ± 8 | 136 ± 14 | 138 ± 15 | 144 ± 15 | 170 ± 11 | 165 ± 14 | 169 ± 10 |
|  | 3 | 121 ± 2 | 130 ± 2 | 140 ± 2 | 134 ± 19 | 137 ± 17 | 139 ± 14 | 160 ± 17 | 167 ± 15 | 163 ± 10 |
| Mesocycle 3 | 1 | 165 ± 2 | 171 ± 2 | 175 ± 2 | 156 ± 14 | 150 ± 16 | 154 ± 18 | 176 ± 9 | 175 ± 16 | 173 ± 14 |
|  | 2 | 159 ± 7 | 166 ± 6 | 176 ± 2 | 136 ± 13 | 140 ± 11 | 138 ± 11 | 164 ± 12 | 169 ± 14 | 168 ± 12 |
|  | 3 | 138 ± 12 | 145 ± 11 | 153 ± 9 | 134 ± 11 | 142 ± 10 | 146 ± 11 | 172 ± 15 | 176 ± 15 | 179 ± 9 |
| Mesocycle 4 | 1 | 168 ± 2 | 174 ± 3 | 179 ± 5 | 146 ± 13 | 147 ± 18 | 152 ± 19 | 169 ± 13 | 169 ± 12 | 171 ± 15 |
|  | 2 | 162 ± 2 | 169 ± 2 | 175 ± 2 | 138 ± 14 | 143 ± 12 | 146 ± 17 | 171 ± 8 | 173 ± 12 | 171 ± 15 |
|  | 3 | 144 ± 2 | 151 ± 2 | 157 ± 2 | 140 ± 18 | 144 ± 16 | 149 ± 13 | 176 ± 15 | 175 ± 14 | 179 ± 10 |
